# Using Automated Live Cell Imaging to Reveal Early Changes during Human Motor Neuron Degeneration

**DOI:** 10.1523/ENEURO.0001-18.2018

**Published:** 2018-06-29

**Authors:** Hye Young Shin, Kathleen L. Pfaff, Lance S. Davidow, Chicheng Sun, Takayuki Uozumi, Fumiki Yanagawa, Yoichi Yamazaki, Yasujiro Kiyota, Lee L. Rubin

**Affiliations:** 1 Department of Stem Cell and Regenerative Biology and Harvard Stem Cell Institute, Harvard University, Cambridge, MA, United States; 2 Healthcare Business Unit, Nikon Corporation, Tokyo, Japan

**Keywords:** Automated live time-lapse imaging instrument, heterogeneity, live cell time-lapse imaging, morphometric analysis, single cell tracking

## Abstract

Human neurons expressing mutations associated with neurodegenerative disease are becoming more widely available. Hence, developing assays capable of accurately detecting changes that occur early in the disease process and identifying therapeutics able to slow these changes should become ever more important. Using automated live-cell imaging, we studied human motor neurons in the process of dying following neurotrophic factor withdrawal. We tracked different neuronal features, including cell body size, neurite length, and number of nodes. In particular, measuring the number of nodes in individual neurons proved to be an accurate predictor of relative health. Importantly, intermediate phenotypes were defined and could be used to distinguish between agents that could fully restore neurons and neurites and those only capable of maintaining neuronal cell bodies. Application of live-cell imaging to disease modeling has the potential to uncover new classes of therapeutic molecules that intervene early in disease progression.

## Visual Abstract

**Figure:**
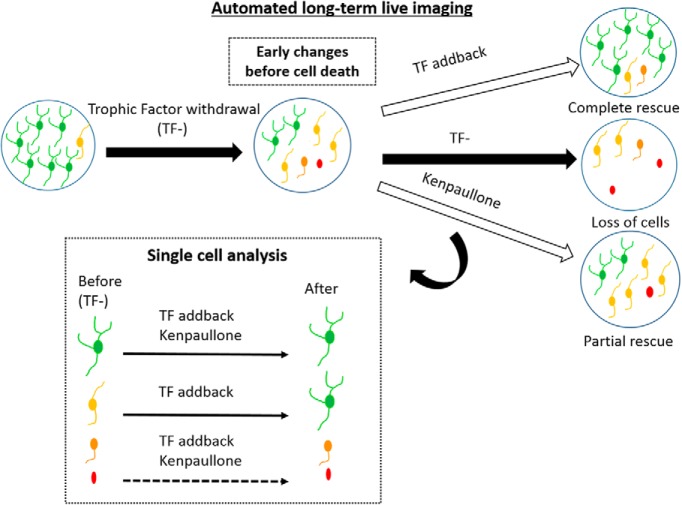

## Significance Statement

This study establishes a new automated live-cell imaging method that was used to analyze human motor neurons in the process of dying after trophic factor withdrawal. Our aim was to provide an alternative to traditional endpoint survival assays by determining morphologic changes that predate death. This was accomplished by tracking large numbers of individual motor neurons over long periods of time. We identified features of motor neurons that distinguish between those neurons that can fully recover from the early stages of degeneration and those that are committed to death. Our research is of clear interest to neuroscientists interested in disease and improved methods for discovering effective therapeutics.

## Introduction

Technical advances in the use of human embryonic stem cell (hESC) and induced pluripotent stem cell (iPSC) technology now allow the production of human cells, especially discrete populations of neurons, for use in disease modeling, regenerative therapy, and drug screening (Grskovic et al., 2011). When compared to either standard transgenic mouse models or postmortem human brains, this provides an unprecedented opportunity to study neurodegenerative diseases such as Alzheimer’s disease (AD), Parkinson’s disease (PD) and amyotrophic lateral sclerosis (ALS; Brennand et al., 2015).

In some recent studies on ALS, survival assays using *in vitro*–derived motor neurons (MNs) from mouse ESCs, hESCs, and patient-derived iPSCs have been employed productively (Yang et al., 2013; Rodriguez-Muela et al., 2017). Nonetheless, these studies present their own sets of challenges. Cell death assays are generally simple, fixed time point counts of cell number, but current differentiation methods often result in heterogeneous mixtures of neuronal and nonneuronal cell types, each with a variable duration of cell survival. Thus, endpoint measurements, which typically measure average changes at a single time point in heterogeneous populations, do not capture the full range of complex biological responses in cells (Arrasate and Finkbeiner, 2005; Skylaki et al., 2016; Rodriguez-Muela et al., 2017). Furthermore, while it is well known that producing neurons from human pluripotent cells can be time consuming—often a period of months—the amount of time required for diseased neurons to die can also be quite protracted.

Time-lapse imaging of individual live cells offers an exciting alternative to address these limitations (Skibinski and Finkbeiner, 2013; Skylaki et al., 2016). This approach also affords an examination of early disease phenotypes, when therapeutic treatments are most effective in prolonging survival and minimizing disease progression. Neuronal morphology can reflect neuronal function and health. Morphologic changes, such as neuronal shrinkage and dendrite retraction, have been reported in postmortem patient samples of AD, PD, and ALS (Davies et al., 1987; Kiernan and Hudson, 1993; Anglade et al., 1997). In particular, surviving MNs in ALS human postmortem tissues show significantly decreased size compared to MNs in healthy tissue, and cell size positively correlates with the number of surviving MNs (Kiernan and Hudson, 1993). Early changes in the properties of axons and dendrites are also a consistent feature of neurodegenerative diseases (Davies et al., 1987; Kiernan and Hudson, 1993; Anglade et al., 1997). So, we hypothesized that defining the earliest stress or disease-related morphologic changes, such as cell body size, neurite length, and number of nodes (also called roots) in cultured human neurons will guide the development of assays to identify drugs that might have the greatest therapeutic efficacy.

In this study, we defined nodes as the location on the cell body from which the neurites project. We focused on node number as a key parameter for neuronal fitness for both practical and technical reasons. The practical reason was that, as mentioned above, neurodegenerative diseases have varying forms of dendritic pathology including loss of neurites, which would be reflected by decreased number of nodes. Counting and tracking nodes is therefore a surrogate measurement for phenotypes found in pathologic specimens from neurodegenerative disease patients. The technical reason centered on reducing the complexity of analyses that depend on the identification and tracking of individual neurites.

Here, we perform a computational, single-cell analysis of *in vitro*–derived human MNs that were individually observed in large numbers for more than 2 weeks to identify key morphometric changes that occur during the onset and reversal of neuronal degeneration leading to cell death. We employed trophic factor (TF) withdrawal as a well-known stressor to study the early processes that underlie MN death. We also measured to ability of TF addback to rescue neurons at various stages of degeneration. Finally, the effects of kenpaullone, previously identified in a MN survival screen (Yang et al., 2013), were compared with effects of TF addback. We used the BioStation CT, a self-contained unit that includes an incubator, a robotic arm, and a microscope, to study the early processes that underlie MN death. Our work was enabled by the development of imaging software that identifies individual MNs and tracks them over time, even when they shift position.

## Materials and Methods

### Motor neuron production

All experiments with hESCs were reviewed and approved by Harvard University Embryonic Stem Cell Oversight Committee. The H9-Islet1::GFP cell line (female human hESCs) was confirmed to be mycoplasma negative by PCR and ELISA. MNs were differentiated from hESCs and grown on Matrigel (Corning) with mTeSR (Stem Cell Technologies), for 21 days. A two-step “hybrid method” was devised in which cells were first maintained on dishes in 2D and later switched to 3D embryoid bodies (EBs). Multipotent neural stem cells (NSCs) were derived from hESCs in 2D. For 2D culture, Accutase-dissociated single hESCs were seeded at a density of 4 × 10^6^ cells with mTeSR media onto coated 10-cm plates (Corning). Vitronectin (Stem Cell Technologies, 10 µg/ml), laminin (Life Technologies, 10 µg/ml), and fibronectin (BD Biosciences, 10 µg/ml) were used for coating. Neural differentiation was induced with basic fibroblast growth factor (bFGF) and dual SMAD inhibition for 7 days (NIM: DMEM/F12 with N2 (Life Technologies), B27 (Life Technologies), Dorsomorphin (Stemgent, 1 µm)/SB431542 (Stemgent, 1 µm)/bFGF (Life Technologies, 10 ng/ml). Three days after plating, retinoic acid (RA; Sigma Aldrich, 3 µm) and brain-derived neurotrophic factor (BDNF; R&D Systems, 30 ng/ml) were added. Smoothened agonist (SAG 1.3; DNSK International, 1 µm) was added at day 5. For 3D cultures, day 8 NSCs were dissociated with Accutase and cultured at a density of 400,000/ml on low-adherence flasks in suspension medium containing ROCK inhibitor (10 µm) and bFGF (10 ng/ml) to generate EBs. Spherical aggregates of NSCs were maintained up to day 21 with RA (1 µm), SAG 1.3 (1 µm), BDNF (10 ng/ml), glial cell–derived neurotrophic factor (GDNF; R&D Systems, 10 ng/ml), insulin-like growth factor (IGF; R&D Systems, 10 ng/ml), cAMP (Santa Cruz Biotechnology, 0.1 µm), and dATP (Santa Cruz Biotechnology, 1 µm). AraC (Sigma Aldrich, 2 µm) was added from day 17 to 21 to eliminate proliferating cells.

**Figure 1.**
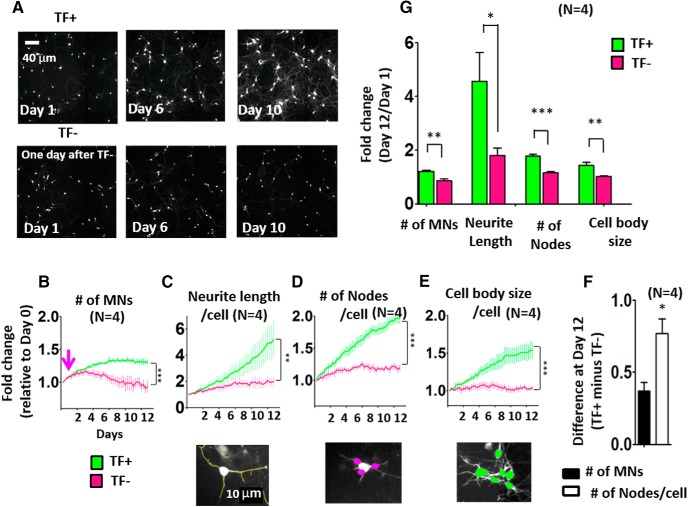
Identifying morphologic changes that precede neuronal death using computational quantitation. ***A***, Representative BioStation CT images of human Islet1::GFP-positive MNs in basal (TF^+^) and withdrawal (TF^–^) conditions at key experimental time points: day 1 (1 day after TF withdrawal), day 6, and day 10 of imaging (scale bar = 40 µm). ***B–E***, Time series plots comparing the populations of Islet1::GFP MNs in TF^+^ (green) and TF^–^ (magenta) conditions. Quantitation was done at each time point for the number (#) of MNs (***B***), neurite length per cell (***C***), number of nodes per cell (***D***), and cell body size per cell (***E***). The arrow indicates time of TF withdrawal (day 1). The average fold change relative to day 0 for all of the measured parameters is shown. A significant different between TF^+^ and TF^–^ over time was found by two-way repeated-measures ANOVA after Bonferroni correction in the number of MNs (*p* < 0.001, *F* = 13.63, DFn = 1; ***B***), neurite length per cell (*p* < 0.01, *F* = 5.27, DFn = 1; ***C***), number of nodes per cell (*p* < 0.001, *F* = 30.57, DFn = 1; ***D***) and cell body size per cell (*p* < 0.001, *F* = 11.54, DFn = 1; ***E***), and significant differences between TF^+^ and TF^–^ were observed at the end point by Bonferroni post-tests: **, *p* < 0.01; ***, *p* < 0.001. Data presented as mean + SEM. (*n* = 4 biological replicate experiments, each with three technical replicates.) ***F***, Comparison of the effect magnitudes between TF^+^ and TF^–^ for number of MNs (***B***) and number of nodes per cell (***D***) at day 12. Data presented as mean + SEM. *, *p* < 0.05 by *t* test (*n* = 4 biological replicate experiments, each with three technical replicates). ***G***, Extracted time series data showing fold change measurements in features between day 1 and day 12. Data presented as mean + SEM. *, *p* < 0.05; **, *p* < 0.01; ***, *p* < 0.001 by *t* test (*n* = 4 biological replicate experiments, each with three technical replicates).

**Figure 2.**
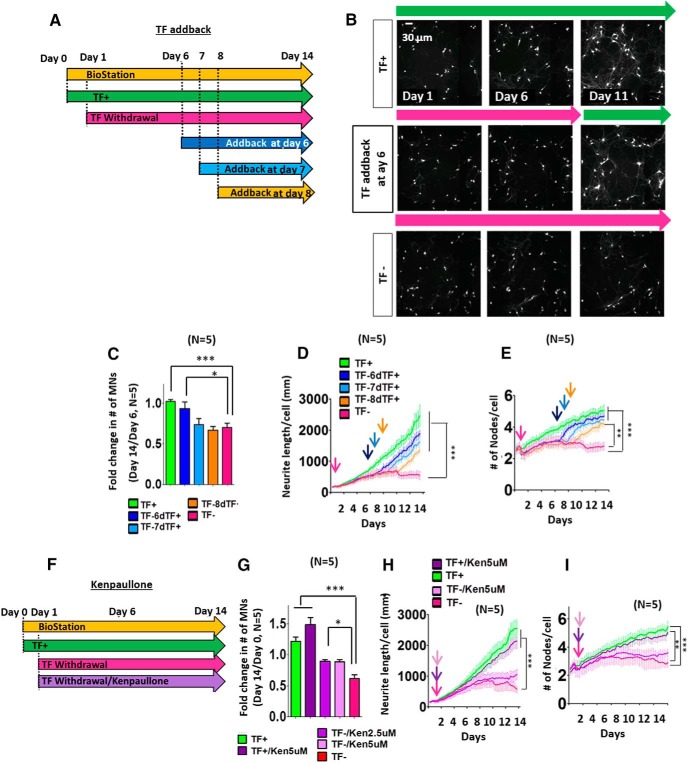
Measuring MN rescue responses following TF addback or kenpaullone treatment. ***A***, Experimental design of TF withdrawal and addback imaging assays. For all experiments, TF withdrawal began 1 day after the start of imaging defined as day 0. For TF addback, the TFs- BDNF, GDNF, and CNTF^–^ were reinstated to the culture media after 6, 7, or 8 days of TF withdrawal. ***B***, Representative images of Islet1::GFP MNs at day 1, 6, and 11 in TF^+^, TF addback at day 6, and TF^–^. ***C***, Fold change in number (#) of MNs between day 6 and day 14 following TF addback at day 6, 7, or 8. All data presented as mean + SEM. *, *p* < 0.05; ***, *p* < 0.001 by *t* test; all compared to TF^–^ conditions (*n* = 5 biological replicate experiments, each with three technical replicates). ***D***, ***E***, Time series plots depicting neurite length (***D***) and number of nodes per cell (***E***) show the MN population behavior in TF^–^ and TF addback conditions. Key experimental time points are denoted with arrows: the start of TF withdrawal (red), day 6 addback (dark blue), day 7 addback (light blue), and day 8 addback (orange). Two-way repeated-measures ANOVA showed significant differences in neurite length per cell (*p* < 0.001, *F* = 6.555, DF*n* = 4; ***D***) and number of nodes (*p* < 0.05, *F* = 3.356, DF*n* = 4; ***E***) over time under the different conditions. Subsequent Bonferroni post tests indicated TF addback at day 6, 7, and 8 increased neurite length per cell (***D***) and the number of nodes (***E***) per cell compared to TF^–^ at the end point. All data presented as mean + SEM. **, *p* < 0.01; ***, *p* < 0.001 by two-way repeated-measures ANOVA with Bonferroni correction, all compared to TF^–^ conditions (*n* = 5 biological replicate experiments, each with three technical replicates). ***F***, Experimental design of imaging assays kenpaullone addition to cells undergoing TF withdrawal. Analysis window between day 6 and day 14 was used for both evaluation of survival effect (***G***) and temporal quantitation of morphometric parameters (***H***, ***I***). ***G***, Fold change in # of MNs between day 0 and day 14 of kenpaullone treatment. All data presented as mean + SEM. *, *p* < 0.05; ***, *p* < 0.001 by *t* test; all compared to TF^–^ conditions (*n* = 5 biological replicate experiments, each with three technical replicates). ***H***, ***I***, Time series plots depicting neurite length (***H***) and number of nodes per cell (***I***) after kenpaullone treatment. Kenpaullone (light and dark purple) was administered from the start of TF withdrawal (magenta) as denoted with arrows. All data presented as mean + SEM. **, *p* < 0.01; ***, *p* < 0.001 by two-way repeated-measures ANOVA with Bonferroni correction, all compared to TF^–^ conditions (*n* = 5 biological replicate experiments, each with three technical replicates).

**Figure 3.**
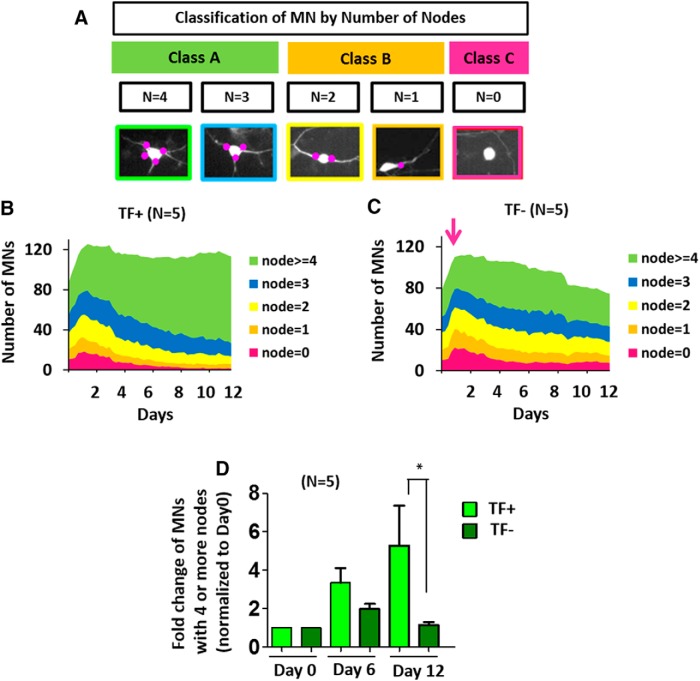
Classifying MNs according to their number of nodes. ***A***, Classification of the heterogeneous MN population according to their number of nodes (0, 1, 2, 3 or 4+ nodes) determines the class assignment (class A, B, C) for each MN. ***B***, ***C***, A stacked temporal area plot displaying the number of MNs with 0, 1, 2, 3 or 4+ nodes, quantified at each time point throughout the imaging experiment. In this stacked area graph, the height of each colored region on the y-axis indicates the number of MNs in each number of nodes category. (***B***), After the first few days in TF^+^, the total number of MNs remains relatively constant. MNs mature over time and eventually MNs with 4+ nodes become the majority of the population. (***C***), In TF^–^, the total number of MNs gradually decreases over time and the MN population comprises a large percentage of cells with 2 or 3 nodes and relatively fewer neurons with 4 or more nodes. The magenta arrow indicates the time of TF withdrawal. ***D***, A histogram of fold change (relative to day 0) in the number of MNs with 4 or more nodes in the TF^+^ and TF^–^ conditions respectively. During the time period, the number of MNs with 4 or more nodes in TF^+^ and TF^–^ conditions becomes increasingly different (*p* < 0.05, *F* = 3.949, DFn = 2 by two-way repeated-measures ANOVA with Bonferroni correction). All data presented as mean + SEM. *, *p* < 0.05. (*n* = 5 biological replicate experiments, each with three technical replicates.)

**Figure 4.**
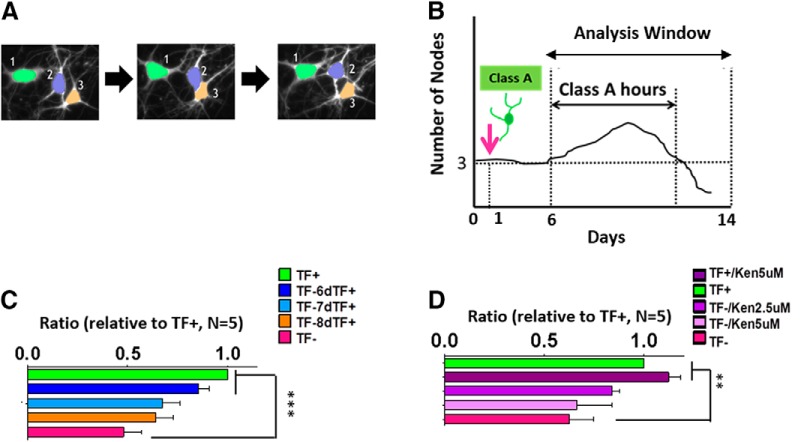
A Single-cell tracking algorithm to measure the lifespan of MNs. ***A***, Representative images of cell tracking. Three MNs distinguished with different colors (green, purple, orange) were tracked by time lapse imaging using CL-Quant software The software was able to assign individual identities to two cells that were overlapping (middle image) using cell body size and position information collected over time. ***B***, Schematic diagram of the class A hours measurement. Class A MNs were defined at the beginning of the imaging period and then tracked. The same analysis window (between day 6 and day 14) was used for all experiments. Using single-cell tracking with a mask for node number, the lifespan hours of class A MNs were measured and averaged during the selected analysis window. For each MN, class A hours ended when class A MNs changed to class B or C. ***C***, ***D***, Normalized class A hours (relative to TF^+^) for TF addback (***C***) and kenpaullone (***D***). Table 1 lists the number of tracked MNs for the TF addback experiments, and Table 2 provides the numbers of tracked MNs for the kenpaullone experiments. Data presented as mean + SEM. **, *p* < 0.01; ***, *p* < 0.001 by *t* test all compared to TF^–^ (*n* = 5 biological replicate experiments, each with three technical replicates).

**Figure 5.**
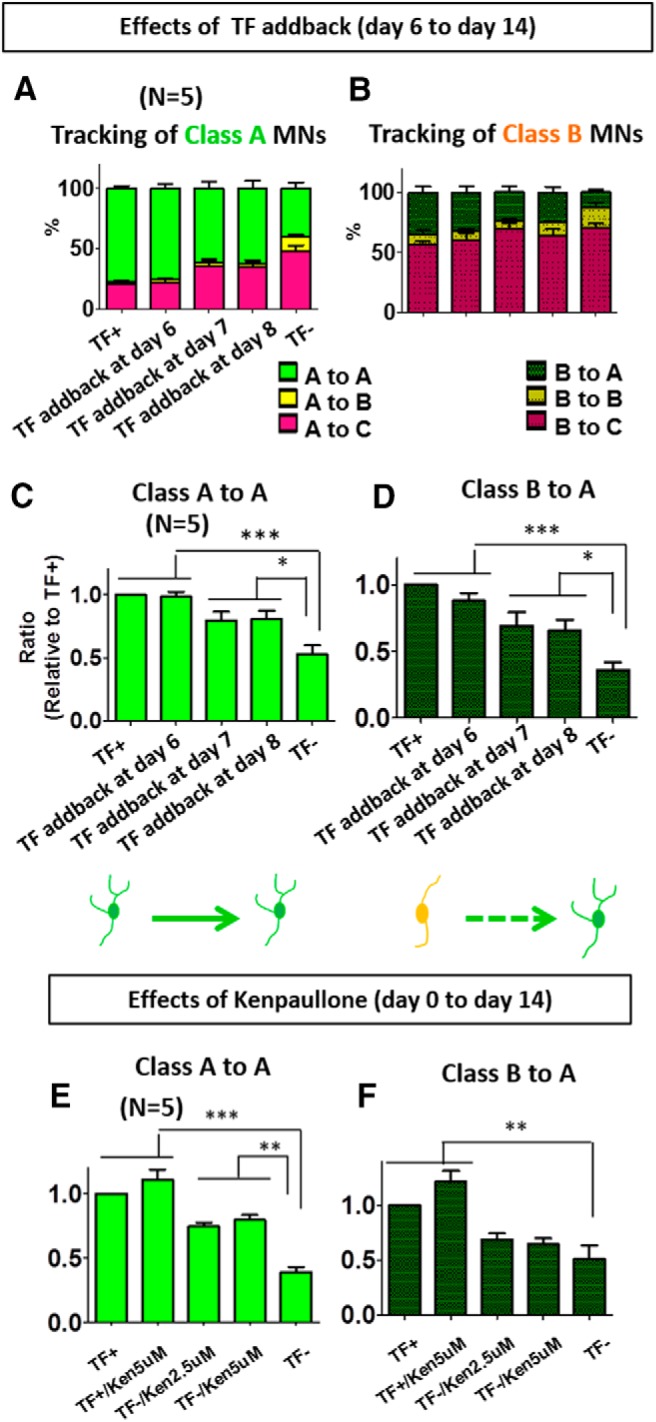
Tracking cell class transitions of individual MNs in TF withdrawal, TF addback, and kenpaullone conditions. Cells were categorized as either class A or class B MNs as shown in Fig. 3*A* and then individually tracked to determine if they remained in the same class at the end of the analysis window. Table 1 details the class transitions for all tracked MNs in the TF addback experiments, while Table 2 provides this information for the kenpaullone experiments. ***A***, MNs identified as class A on day 6 were tracked until day 14 in each treatment condition and were categorized according to their class transition in a stacked histogram (*n* = 5 biological replicate experiments, each with three technical replicates). ***B***, Stacked histogram of class transitions from day 6 to day 14 of class B MNs in each treatment condition (*n* = 5 biological replicate experiments, each with three technical replicates). ***C***, The ratio of class A to A MNs (from day 6 to day 14) in each different treatment condition, relative to TF^+^ control, quantifies the maintenance of class A MNs. TF addback at day 6 shows a significant rescue effect, while TF addback at day 7 or 8 is less effective. Data presented as mean + SEM. *, *p* < 0.05; ***, *p* < 0.001 by *t* test; all compared to TF^–^ (*n* = 5 biological replicate experiments, each with three technical replicates). ***D***, The ratio of class B to A MNs (from day 6 to day 14) in the different treatment conditions relative to TF^+^ quantifies the rescue of class B MNs to class A MNs. Data presented as mean + SEM. *, *p* < 0.05; ***, *p* < 0.001 by *t* test; all compared to TF^–^ (*n* = 5 biological replicate experiments, each with three technical replicates). ***E***, Maintenance of class A to A MNs from day 0 to day 14 in different treatment conditions relative to TF^+^. Kenpaullone significantly maintained class A MNs as class A compared to TF withdrawal. Data presented as mean + SEM. **, *p* < 0.01; ***, *p* < 0.001 by *t* test; all compared with TF^–^ conditions (*n* = 5 biological replicate experiments, each with three technical replicates). ***F***, Changes in the ratio of class B to A MNs from day 0 to day 14. Kenpaullone did not rescue many class B MNs to class A. Data presented as mean + SEM. **, *p* < 0.01; ***, *p* < 0.001 by *t* test; all compared with TF^–^ conditions (*n* = 5 biological replicate experiments, each with three technical replicates).

**Figure 6.**
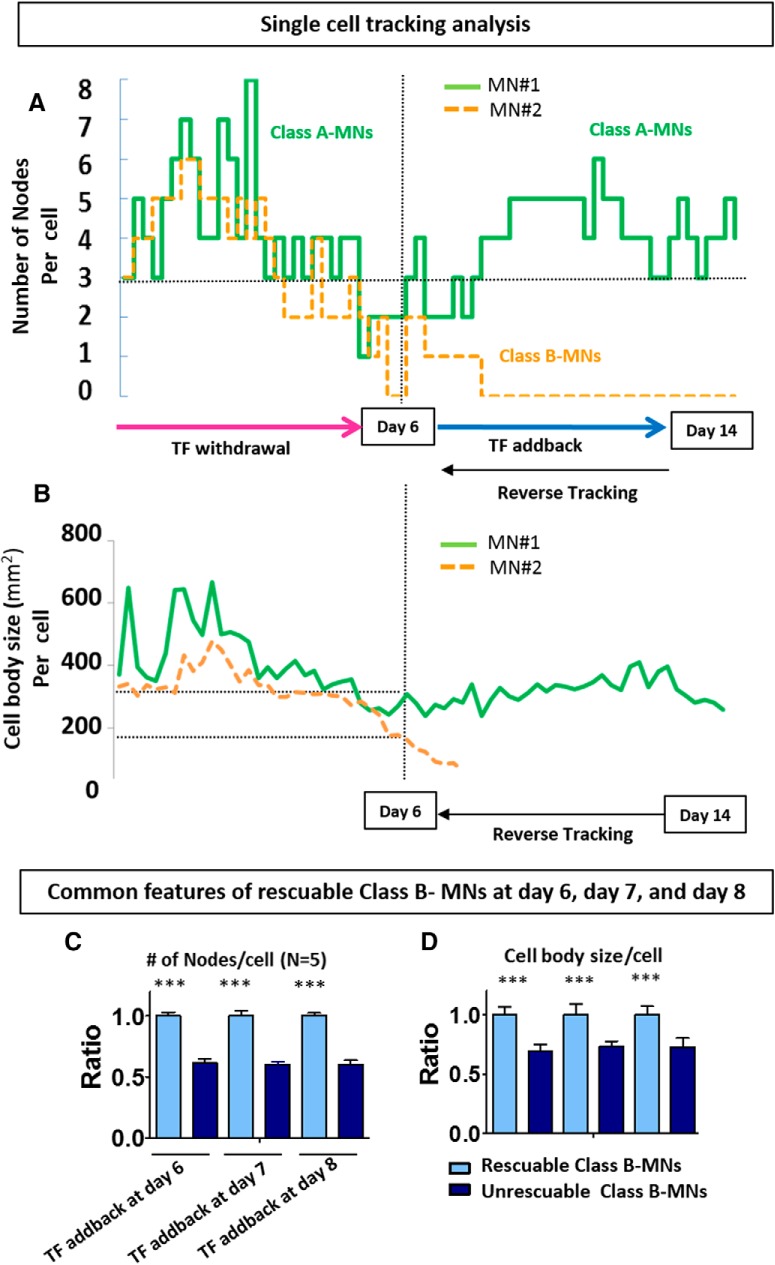
Characterization of key morphologic features of rescuable class B MNs using reverse tracking. ***A***, ***B***, Representative single cell analysis extracted using CL-Quant. Graphs show tracked number of nodes (***A***) and cell body size (***B***) of two individual MNs over time. In this plot, both MN #1 and MN #2 became class B MNs after TF withdrawal at day 6. MN #1 recovered to class A following day 6 of TF addback. ***C***, ***D***, Common features of rescuable class B MNs at day 6, 7 and 8. At the time point immediately before TF addback, class B MNs that could develop into class A MNs had more nodes (***C***) and a larger cell body size (***D***) compared to class B MNs that could not regrow neurites. Data presented as mean + SEM. ***, *p* < 0.001 by *t* test; all compared with TF^–^conditions (*n* = 5 biological replicate experiments, each with three technical replicates).

In this study, assay development was necessary to determine the optimal cell density to achieve uniform distribution of MNs. We tested the robustness of the experimental system across multiple biological replicates using independent batches of MNs. There is some interexperimental variability, which is a combined consequence of variability in the differentiation efficiency, differential cell survival during dissociation, and deviations in the plating cell densities. Despite this, four (Fig. 1) and five (Figs. 2–6) independent batches of MN differentiation exhibited remarkably consistent trends in analyzed results, indicating that this model is reliable and reproducible.

### Treatment of cells

Withdrawal of trophic factors [TFs; BDNF, GDNF, and ciliary neurotrophic factor (CNTF)] is a well-established method to activate neuronal apoptosis (Yang et al., 2013). To initiate cell death in our cultures, we withdrew TF support along with B27 and N2 supplements from MNs (TF^–^) at day 1 (1 day after live imaging initiation). To study the early processes that underlie MN death by TF withdrawal, as well as to distinguish different actions of kenpaullone and TF addback treatment on the MNs deprived of TF at day 1, TFs (BDNF, GDNF, CNTF, B27, and N2) were reintroduced to the cultures (defined as “TF addback”) at varying lengths of time (6, 7, or 8 days) after their withdrawal. For kenpaullone treatment, two different concentrations (2.5 and 5 µm) were supplied to MNs during the entire period in which they were maintained in the absence of TF.

### Assay development for automated live time-lapse imaging

To prepare MNs for live imaging, day 21 EBs were dissociated with Accutase, triturated until no clumps were visible, and seeded into 96-well µClear black-walled plates (Greiner Bio-One; Cat # 655090) with primary mouse glia as feeder cells, and maintained with BDNF (10 ng/ml), GDNF (10 ng/ml), and CNTF (10 ng/ml). FluoroBrite DMEM (Thermo Fisher Scientific) medium with N2 and B27 was used to minimize background fluorescence.

Cell density is a critical variable in neuronal cultures and for *in vitro* live-cell imaging. We first evaluated how MNs plated at different initial densities performed in our survival assay. We compared cells plated at 3.5 × 10^4^ (35K) cells and at 6.0 × 10^4^ (60K) cells per well. We tracked an average of 110 individual GFP-positive MNs in the 35K/well plating density and 140 MNs per well in the 60K/well plating density (data not shown). We noted that the number of MNs that were able to be tracked did not increase linearly with the number of cells plated. This reflects an inherent challenge with automated live cell tracking, which is that cells are more difficult to individually distinguish and track as they become more crowded. When plating at a lower cell density onto a glial support layer, we observed that MNs exhibit little clumping, which yields a single neuron distribution for more accurate tracking and measurements. Therefore, a 35K/well plating density was employed for all subsequent experiments.

### Automated time-lapse live imaging

Three technical replicate wells per condition per experiment (four biological replicates for Fig. 1; five biological replicates for Figs. 2–6) were imaged. A 10× objective was used to acquire phase and fluorescent images at each time point. For each well, a 4 × 4 stitched tiling captured an area of 3.08 × 3.08 mm, which covers ∼30% of the total growth area of the well. Images were automatically captured every 6 hours for 14–17 days.

### Cell tracking and image analysis

All image analysis was performed using CL-Quant software (Nikon Corporation), and Nikon designed specialized viewer software to easily review the analysis results from these data sets. To detect the morphologic properties of the cells in the culture, morphologic filters (or masks) were designed to identify and quantify the different features, including cell body number, neurite length, cell body size, compactness, and intensity, in the raw image. To detect nodes, a combination of the cell body filter and neurite filter was used to detect the overlapping regions between neurite and cell body. This filter combination was then used to identify nodes in the raw images. After the filters for detection of each cell were optimized, the tracking was performed using the filter for cell body. The setting parameters used in CL-Quant for this tracking are as follows: minimum object size: 50, maximum object size: 999999, maximum search range: 200, split threshold: 0.90, merge threshold: 0.70, minimum trajectory length: 5, object split: ignore split, object merge: merge with partition, enable lineage: off, enable robust measurement: off, object-to-object overlap: off, remove short track when merging: off.

To track individual cells, a cell ID number was assigned to each identified cell body at the beginning of the imaging period. Comparing the cell body filters between two subsequent time frames for morphology, position, and overlap, scores were given to allow the best assignment of cell IDs (see example images in Fig. 4*A*). This computational matching was performed iteratively throughout the entire time-lapse data set. By joining and tracking the cell IDs with the highest score, cell tracking was completed for all objects identified by the cell body filter.

The tracking data set for the cell body filter was then used as the basis to track the number of nodes over time. A moving average (MA) was employed for smoothing the data set before plotting. The node detection algorithm was further used to define class A MNs (with three or more nodes) and class B MNs (with one or two nodes) and to individually track MNs to determine survival outcomes. The number of MNs tracked for all TF addback and kenpaullone experiments at the 35K seeding density can be found in Tables 1 and 2.

**Table 1.** Numbers of tracked MNs to evaluate cell class transitions following TF addback (day 6–14)

			Outcome of fate transition				Outcome of fate transition		
Conditions	Total tracked # of MNs	Tracked # of Class A MNs	Class A to A	Class A to B	Class A to C	Tracked # of Class B MNs	Class B to A	Class B to B	Class B to C
TF^+^	110.9 ± 32.05	83.33 ± 30.16	62.27 ± 22.07	2.267 ± 1.024	18.80 ± 7.240	27.57 ± 4.540	9.533 ± 2.421	3.300 ± 0.9638	14.73 ± 2.379
TF addback at day 6	93.60 ± 29.36	58.33 ± 24.68	40.17 ± 16.36	2.033 ± 1.311	16.13 ± 7.285	35.27 ± 6.954	10.43 ± 2.834	3.167 ± 0.7853	21.67 ± 4.001
TF addback at day 7	97.97 ± 28.09	46.92 ± 16.25	26.56 ± 11.81	1.333 ± 0.5164	19.03 ± 4.254	44.67 ± 11.90	33.90 ± 17.07	2.100 ± 0.9422	21.27 ± 6.517
TF addback at day 8	110.3 ± 31.51	57.27 ± 24.46	33.90 ± 17.07	2.100 ± 0.9422	21.27 ± 6.517	55.54 ± 11.60	25.83 ± 13.03	5.600 ± 1.431	24.43 ± 6.128
TF^–^	101.6 ± 30.11	55.87 ± 19.52	25.83 ± 13.03	5.600 ± 1.431	24.43 ± 6.128	45.73 ± 12.47	5.267 ± 2.125	10.70 ± 4.803	29.77 ± 6.136

Data are presented as mean ± SEM (*n* = 5).

**Table 2.** Numbers of tracked MNs to evaluate cell class transitions during kenpaullone treatment (day 0–14)

			Outcome of fate transition				Outcome of fate transition		
Conditions	Total tracked # of MNs	Tracked # of Class A MNs	Class A to A	Class A to B	Class A to C	Tracked # of Class B MNs	Class B to A	Class B to B	Class B to C
TF^+^	86.20 ± 17.08	42.47 ± 13.86	27.47 ± 10.07	0.6000 ± 0.2449	14.40 ± 4.128	43.73 ± 6.322	14.87 ± 4.121	1.733 ± 0.5907	27.13 ± 3.023
TF^+^/Ken 5uM	80.27 ± 19.01	41.07 ± 15.52	27.60 ± 11.26	0.7333 ± 0.3399	12.73 ± 4.153	39.20 ± 6.538	17.00 ± 4.791	2.333 ± 0.5055	19.87 ± 2.277
TF^–^/Ken 2.5uM	98.27 ± 21.23	42.80 ± 16.05	21.40 ± 8.362	2.400 ± 0.8781	19.00 ± 7.551	55.47 ± 10.46	15.07 ± 4.993	7.000 ± 1.623	33.40 ± 5.499
TF^–^/Ken 5uM	96.87 ± 16.16	38.27 ± 11.93	18.67 ± 5.544	1.867 ± 0.7196	17.73 ± 5.743	58.60 ± 12.21	11.80 ± 4.196	7.200 ± 2.560	39.60 ± 7.318
TF^–^	92.53 ± 19.25	42.47 ± 14.90	11.13 ± 3.868	4.467 ± 1.698	26.87 ± 9.443	50.07 ± 6.998	6.867 ± 1.695	4.333 ± 1.038	38.87 ± 5.904

Data are presented as mean ± SEM (*n* = 5).

When it was observed that TF addback rescued some class B MNs to class A MNs, we devised a reverse tracking approach to distinguish rescuable class B MNs from unrescuable class B MNs. Rescued and unrescued class B MNs were identified and individually tracked backward to the time point of addback (day 6, 7, or 8). Then, morphologic parameters such as node number, cell body size, compactness, and velocity at days 6, 7, and 8 were tallied and averaged.

### Statistical analysis

Statistical analysis was performed using GraphPad Prism 5. All data (four biological replicates: Fig. 1; five biological replicates: Figs. 2–6) are presented as the mean + SEM. The experiments were not randomized. The scientists were not blinded during experiments; however, the imaging analysis was conducted in an unbiased way across all samples and was based on automated image analyses. Repeated measure–based parameters (such as neurite length per cell, number of nodes, and cell body size per cell over time) were analyzed using two-way repeated-measures ANOVA followed by Bonferroni correction, followed by *t* test. Statistical details (*p* value, *F*, and degree of freedom (Dfn) are provided in Table 3 along with the results of the two-way ANOVA testing. When only two groups were analyzed, statistical significance was determined using unpaired two-tailed *t* test. Normal (Gaussian) distribution was determined by column test. A *p* value of <0.05 (*), <0.01 (**), and <0.001(***) was used to denote statistical significance. Superscript letters listed with figure legends and *p*-values correspond to the statistical tests shown in Table 3.

**Table 3.** Summary of statistical analyses

Location	Description	Data structure	Type of test	Statistical value
a	Fig. 1*B*–*E*	Normal (Gaussian distribution) at the end point; number (*n*) of biological experiments = 4 with three technical replicates	Two-way repeated-measures ANOVA after Bonferroni correction	Number (#) of MNs : *p* < 0.001, *F* = 13.63, DFn = 1; Neurite length per cell: *p* < 0.01, *F* = 5.27, DFn = 1; # of Nodes per cell : *p* < 0.001, *F*= 30.57, DFn = 1; Cell body size per cell : *p* < 0.001, *F* = 11.54, DFn = 1
			Unpaired two-tailed *t*-test at the end point	# of MNs : *p* < 0.001; Neurite length per cell: *p* < 0.01; # of Nodes per cell : *p* < 0.001; Cell body size per cell : *p* < 0.001
b	Fig. 1*F*	Normal, *n* = 4	Unpaired two-tailed *t*-test	*p <* 0.05
c	Fig. 1*G*	Normal, *n* = 4	Unpaired two-tailed *t*-test	# of MNs: *p* < 0.01, Neurite length per cell: *p* < 0.05; # of Nodes per cell: *p* < 0.0001, Cell body size: *p* < 0.01
d	Fig. 2C	Normal, *n* = 5	Unpaired two-tailed *t*-test	TF^+^: *p* < 0.001, TF addback at day 6: *p* < 0.05
e	Fig. 2D, *E*	Normal at the end point; *n* = 5	Two-way repeated-measures ANOVA after Bonferroni correction	Neurite length per cell: *p* < 0.01, *F* = 6.555, DFn = 4; # of Nodes per cell: *p* < 0.05, *F* = 3.356, DFn = 4
			Unpaired two-tailed *t*-test at the end point	Neurite length per cell: TF addback at day 6, day 7, and day 8: *p* < 0.001, respectively; # of Nodes per cell: TF addback at day 6, day 7: *p* < 0.001; TF addback at day 8: *p* < 0.01
f	Fig. 2G	Normal, *n* = 5	Unpaired two-tailed *t*-test	TF^+^ and TF^+^/Ken 5µM: *p* < 0.001; TF^–^/2.5µM and TF^–^/5µM: p < 0.05
g	Fig. 2*H, I*	Normal at the end point; *n* = 5	Two-way repeated-measures ANOVA after Bonferroni correction	Neurite length per cell: *p* < 0.05, *F* = 4.01, DFn = 4; # of Nodes per cell: not significant
			Unpaired two-tailed *t*-test at the end point	Neurite length per cell: TF^+^ and TF^+^/Ken 5µM: *p* < 0.001; # of Nodes per cell: TF^+^: *p* < 0.001,; TF^+^/Ken5 µM: *p* < 0.01
h	Fig. 3D	Normal at the end point; *n* = 5	Two-way repeated-measures ANOVA correction after Bonferroni correction	Fold change of MNs with 4 or more nodes :*p* < 0.05, *F* = 3.949, DFn = 2
			Unpaired two-tailed *t*-test at the end point	*p* < 0.05
i	Fig. 4C	Normal, *n* = 5	Unpaired two-tailed *t*-test	TF^+^: *p* < 0.001, TF addback at day 6: *p* < 0.001
j	Fig. 4D	Normal, *n* = 5	Unpaired two-tailed *t*-test	TF^+^: *p* < 0.01, TF^+^/Ken 5 µM: *p* < 0.01
k	Fig. 5C	Normal, *n* = 5	Unpaired two-tailed *t*-test	TF^+^: *p* < 0.001, TF addback at day 6: *p* < 0.001, TF addback at day 7: *p* < 0.05, addback at day 8: *p* < 0.05,
l	Fig. 5D	Normal, *n* = 5	Unpaired two-tailed *t*-test	TF^+^: *p* < 0.001, TF addback at day6: *p* < 0.001,; TF addback at day7: *p* < 0.05, addback at day8: *p* < 0.05
m	Fig. 5E	Normal, *n* = 5	Unpaired two-tailed *t*-test	TF^+^: *p* < 0.001, TF^+^/Ken 5 µM: *p* < 0.001,; TF^–^/Ken 2.5 µM: *p* < 0.01, TF^–^/Ken 5µM: *p* < 0.01
n	Fig. 5F	Normal, *n* = 5	Unpaired two-tailed *t*-test	TF^+^: *p* < 0.01, TF^+^/Ken 5 µM: *p* < 0.01
o	Fig. 6C	Normal, *n* = 5	Unpaired two-tailed *t*-test	TF addback at day 6, day 7, and day 8: *p* < 0.001, respectively
p	Fig. 6D	Normal, *n* = 5	Unpaired two-tailed *t*-test	TF addback at day 6, day 7, and day 8: *p* < 0.001, respectively

## Results

### Assay development for automated robotic live-cell imaging of MN cultures

For experiments described here, we took advantage of the fact that there are numerous protocols available for deriving human and mouse MNs from ESCs or iPSCs, and we sought to identify the most robust differentiation method for our live-cell imaging assay. Specifically, we used an MN reporter hESC line (H9-Islet1::GFP), in which GFP expression is regulated by Islet1, an MN-specific transcription factor (Rigamonti et al., 2016). Preliminary experiments were performed to establish reproducible conditions for live-cell imaging. We tested several conditions including different MN differentiation protocols, seeding densities, and plating conditions. We selected a modified MN differentiation protocol (Yang et al., 2013; Qu et al., 2014), and plated MNs on astrocytes to prevent cells from clumping, which interfered with our ability to track single cells. To achieve high-resolution imaging without phototoxicity, we evaluated different illumination intensities and exposure times and used FluoroBrite DMEM medium to reduce background fluorescence. To initiate cell death in our cultures, we withdrew TFs (BDNF, GDNF, and CNTF) from culture medium. This is a well-established method to activate and study neuronal apoptosis (Yang et al., 2013). We used time-lapse live imaging to collect data at multiple time points over ∼2 weeks. We then developed algorithms that enabled computational analysis of morphologic changes during the progressive stages of cell death (Videos 1–3).

### Identification of early-onset morphometric changes after TF withdrawal

Neurons have a complex morphology, with many neurites (axons and dendrites) extending from the cell body (Fig. 1*A*). We used computational algorithms to analyze the number of MNs (Fig. 1*B*), the most commonly used survival parameter, and to quantify several key morphologic features, such as neurite length, number of nodes per cell, and cell body size (Fig. 1*C–E*). In the presence of TFs (TF^+^), the number of MNs was relatively unchanged over time (Fig. 1*B*), while neurite length per cell (Fig. 1*C*), number of nodes per cell (Fig. 1*D*), and cell body size per cell (Fig. 1*E*) continued to increase. In contrast, after the removal of TFs (TF^–^), neurite length per cell, number of nodes per cell, and cell body size per cell did not increase, and the number of MNs gradually decreased (Fig. 1*B–E*). Two-way repeated-measures ANOVA showed significant differences between TF^+^ and TF^–^ conditions over time in number of MNs, neurite length per cell, number of nodes per cell, and cell body size per cell over time (Fig. 1*B–E* ^a^, Table 3^a^). Subsequent Bonferroni posttests indicated that all the descriptors have significant differences between TF^+^ and TF^–^ conditions at the endpoint (Fig. 1*B–E*). Differences in the effect magnitudes in the number of nodes was striking compared to that in the number of MNs at day 12 (Fig. 1*F* ^b^).

We measured the fold change in the different morphometric parameters between days 1 and 12. Our results demonstrated that number of nodes per cell exhibited significant differential responses between TF^+^ and TF^–^ conditions (Fig. 1*G* ^c^; *n* = 5, *p* < 0.0001). The greatest absolute difference was observed when measuring neurite length per cell; however, the node number measurement proved to be a more reproducible value between biological replicates and therefore had the lowest *p*-value. For this reason, we concluded that quantifying the number of nodes is a more sensitive indicator of the presence of TF support than measuring MN survival itself.

### Population-based analysis of the protective effects of TFs and kenpaullone

We sought to define critical points in the neuronal degeneration and regeneration processes using population analysis of all cultured cells. We tracked and averaged the number of MNs, as well as their morphometric features, over time following TF withdrawal. We specifically aimed to determine for how long after TF withdrawal were MNs capable of being rescued by restoring TFs to the medium (TF addback), another paradigm that has been used for many years (Edwards et al., 1991). MNs underwent TF withdrawal for 6, 7, or 8 days, after which cells were either replenished with TF or maintained without trophic support (Fig. 2*A, B*). Tracking MN survival over time demonstrated that TF addback at day 6 was still capable of rescuing MNs (Fig. 2*C* ^d^; *n* = 5, *p* < 0.05). At each time after TF withdrawal up to 8 days, TF addback increased neurite length per cell (Fig. 2*D* ^e^; *n* = 5, *p* < 0.001) and number of nodes per cell (Fig. 2*E* ^e^; *n* = 5, TF addback at day 6, day 7: *p* < 0.001; TF addback at day 8: *p* < 0.01), although the extent of recovery decreased with longer periods of TF withdrawal. Importantly, changes in these morphologic properties of MNs were again much more sensitive measures of cellular responses to the presence of TFs than was the number of cell bodies.

We also determined the effects of kenpaullone, an agent previously identified in an MN survival screen (Yang et al., 2013), using MNs deprived of TF. In this case, we treated MNs with two different concentrations of kenpaullone during the entire period in which they were maintained in the absence of TF (Fig. 2*F*). Under these conditions, we found that kenpaullone could preserve many of the MN cell bodies (Fig. 2*G* ^f^; *n* = 5, *p* < 0.05), and, to some degree, neurite length and the number of nodes, although this was not statistically significant (Fig. 2*H*, *I* ^g^; *n* = 5). Video 3 illustrates dynamic single-cell analysis of morphologic changes of MNs in different experimental conditions: TF, TF addback at day 6, 5 µm kenpaullone, and TF withdrawal.

### Single-cell tracking–based parameters to evaluate the protective effects of TFs and kenpaullone

Having quantified the responses of MN populations to varying TF conditions, we wanted to obtain additional information through tracking and quantifying the behaviors of individual cells such as number of nodes, cell body size, compactness, intensity, and velocity of their movement. One reason for doing this is that it has been demonstrated that distinct populations of cultured human MNs are much more prone to death then others (Arrasate and Finkbeiner, 2005; Skylaki et al., 2016; Rodriguez-Muela et al., 2017). First, we developed single-cell tracking metrics defining MN subpopulations according to their number of nodes (Videos 1 and 2). The number-of-nodes metric has several advantages that make it ideal for single-cell tracking. First, it correlates well with overall neuron health, since an increase or decrease in the number of nodes in MN corresponded to both TF withdrawal and rescue responses. Second, the number of nodes could be readily tracked by live-cell imaging at the single-cell level (Video 2). Representative tracking analysis of neurite length (yellow), number of nodes (magenta), and cell body size (green) of MNs in four experimental conditions is shown in Video 3. In TF^+^ (Video 3*A*), individual MNs showed increased neurite length and number of nodes. TF addback at day 6 (Video 3*B*) rescued both neurite length and number of nodes. 5 µm kenpaullone (Video 3*C*) was less protective compared to TF addback (Video 3*B*). After TF withdrawal (Video 3*D*), the neurites retracted and the cell body size and number of nodes decreased.

Video 1. Representative video of H9-Islet1::GFP MN, captured every 6 hours for 14 days. Single-cell tracking software successfully identifies and tracks individual MNs. Green dot indicates center of the cell body. The red line maps the MN’s trajectory over time. 10.1523/ENEURO.0001-18.2018.video.1

Video 2. Morphometric single-cell tracking analysis tracks neurite length (yellow), number of nodes (magenta), and cell body size (green) of MN over time. Because of the increasing complexity of the neuritic network, the number of nodes metric is better than neurite length for tracking at the single-cell level. Images were captured every 6 hours for 14 days. 10.1523/ENEURO.0001-18.2018.video.2

Video 3. Single-cell tracking analysis of neurite length (yellow), number of nodes (magenta), and cell body size (green) of MN in four experimental conditions: TF **(*A*)**, TF addback at day 6 **(*B*)**, 5 µm kenpaullone and TF withdrawal **(*C*)**, and TF withdrawal **(*D*)**. TF was withdrawn at day 1 (1 day after live imaging initiation), and TF was added at day 6 **(*C*)**, and 5 μm kenpaullone was added during the entire period in which MNs were maintained in the absence of TF. Images were captured every 6 hours for 14 days. In TF **(*A*)**, individual MNs increased neurite length and number of nodes, and TF addback at day 6 **(*B*)** rescues both neurite length and number of nodes. Kenpaullone **(*C*)** is less protective compared to TF addback **(*B*)**. In TF withdrawal **(*D*)**, the neurites retract and the cell body size and number of nodes decrease. 10.1523/ENEURO.0001-18.2018.video.3

Thus, we divided MNs into five categories according to their number of nodes (*n* = 0, 1, 2, 3, >4; Fig. 3*A*) and quantified the number of MNs in each category over time (Fig. 3*B*, *C*). Comparing MN compositions between TF^+^ (Fig. 3*B*) and TF^–^ (Fig. 3*C*) conditions revealed striking differences. In TF^+^, MNs with 4 or more nodes were the most abundant population and increased throughout the imaging period, while the number of MNs in the other categories decreased (Fig. 3*B*). This reflects the natural maturation of MNs developing a more complex neurite network. However, in TF^–^, the population of MNs with 4 or more nodes was significantly smaller and did not continue to increase over time (Fig. 3*C*). In the TF^–^ condition, the proportion of each type of MN remained fairly constant throughout, with a slight decrease of MNs in all categories over time, indicating cell death that accompanies TF withdrawal (Fig. 3*C*). The number of MNs with 4 or more nodes became statistically significant between TF^+^ and TF^–^ conditions over time (Fig. 3*D* ^h^; *n* = 5, *p* < 0.05, *F* = 3.949, DFn = 2). At the end of the analysis (day 12), the number of MNs with 4 or more nodes was significantly higher in TF^+^ compared to TF^–^ (Fig. 3*D* ^h^; *n* = 5, *p* < 0.05).

We next collected data on single MNs classified according to their number of nodes: class A (three or more nodes; the most well-developed cells), class B (one or two nodes), and class C (no nodes). We wanted to determine if neurons with more nodes were more likely to survive TF withdrawal than neurons with fewer nodes. We started by individually tracking the MNs with the most nodes (class A). We defined a metric, “class A hours,” as the length of time in which MNs remained as class A (Fig. 4*B*). After TF withdrawal, class A MNs transitioned into class B or C, resulting in a shorter class A hours period. However, TF addback at day 6 significantly increased class A hours (Fig. *4C* ^i^; *n* = 5, *p* < 0.001). TF addback at days 7 and 8 appeared to increased class A hours somewhat as well, but those effects were not statistically significant. Next, we used the same class A hour measurement to evaluate kenpaullone’s effects on MNs, using the same experimental conditions as in Fig. 4*D*. Kenpaullone-treated MNs showed a non–statistically significant trend toward increasing class A hours of MNs undergoing TF withdrawal (Fig. 4*D* ^j^; *n* = 5).

We next sought to examine the morphometric changes of individual MNs over time after TF withdrawal using computational single-cell tracking. We could not track class C MNs, as they quickly died and disappeared from view. Thus, we tracked the individual fate transitions within and between class A (Fig. 5*A*) and class B (Fig. 5*B*) MNs by measuring changes in the number of nodes per cell. We found that two cell fate transitions—class A to A (Fig. 5*C, E*) and class B to A (Fig. 5*F*)—were useful in evaluating MN rescue effects. Most class A MNs remained in class A (class A to A) after TF addback at day 6, 7, and 8 (Fig. 5*C* ^k^; *n* = 5, TF addback at day 6: *p* < 0.001, TF addback at day 7: *p* < 0.05, TF addback at day 8: *p* < 0.05). Similarly, tracking class B MNs indicated that more class B MNs were rescued to class A MNs (class B to A) after TF addback at day 6, 7, and 8 compared to the TF withdrawal condition, with the largest rescue occurring at day 6 (Fig. 5*D* ^l^; TF addback at day 6: *p* < 0.001, TF addback at day 7: *p* < 0.05, addback at day 8: *p* < 0.05). We also tested the effects of kenpaullone under the same conditions as in Fig. 2*F*. Again, we found that this compound could maintain class A MNs (Fig. 5*E* ^m^; *n* = 5, *p* < 0.01), but in contrast to TF addback, kenpaullone did not cause class B MNs to develop into class A MNs (Fig. 5*F* ^n^; *n* = 5). Meanwhile adding kenpaullone at day 6 did not improve survival (data not shown). Tables 1 and 2 show MN numbers for each category.

### Reverse tracking analysis elucidates key features of rescuable class B MNs

Results from the previous section established that many class A MNs could retain class A features after both TF addback and kenpaullone treatment, but only TF could permit class B MNs to mature into class A MNs. However, not all class B MNs were rescued to class A MNs, even after TF addback. Fig. 6*A* and *B* shows representative single-cell analyses for number of nodes and cell body size. Both MN #1 and MN #2 “regressed” into class B after TF withdrawal at day 6, but only MN #1 recovered to class A after TF addback (Fig. 6*A*). We next defined the characteristics of class B MNs that predicted their ability to be fully rescued by TF addback. First, we identified individual class B MNs that either were or were not rescued after TF addback at day 6, 7, or 8. Then, we reverse-tracked the MNs back to the time of TF withdrawal and examined key morphologic parameters, such as number of nodes (Fig. 6*C*), cell body size (Fig. 6*D*), compactness, and velocity (data not shown) at day 6, 7, and 8. This analysis established that the class B MNs that could recover to class A MNs after TF addback had more nodes (Fig. 6*C* ^°^; *n* = 5, *p* < 0.001) and a larger cell body (Fig. 6*D* ^p^; *n* = 5, *p* < 0.001) compared to class B MNs that did not recover. Thus, MNs with more nodes and larger cell bodies are the subset of MNs that are most likely to recover after a death stimulus, whereas class C MNs cannot be rescued with any of the treatments or compounds we used.

## Discussion

Live-cell imaging of individual cells may be much more informative than population-based methods of studying cell behavior (Arrasate and Finkbeiner, 2005; Sharma et al., 2012). This is particularly true in stem cell–derived neuronal cell cultures that typically are quite variable in composition. However, it can be somewhat onerous to carry out and is normally quite limited in terms of the numbers of cells that can be analyzed (Skibinski and Finkbeiner, 2013; Skylaki et al., 2016). In this paper, we used the BioStation CT that was developed to facilitate prolonged visualization of cells. Analysis of cells using this instrument in combination with advanced analysis software provided us with the ability to computationally identify, categorize, and track individual cells. Our work shows that this approach provides detailed measurements of morphologic changes that occur in individual MNs over time as they degenerate.

The software we employed is highly customizable, so any morphologic attribute can be selected to categorize and track cell outcomes. Importantly, these time-dependent measurements do not necessarily need to be predefined. Because each cell is individually tracked, its “lifetime” can be computationally annotated by specific morphologic features. This ability is especially relevant to *in vitro*–derived cultures, because the terminally differentiated cells of interest arise and mature at different times. We used the morphometric analysis assay to detect distinct modes of action underlying MN rescue seen with TF addback versus kenpaullone treatment. While both treatments were efficacious, only TF addback restored class B MNs to class A. In contrast, kenpaullone primarily acted to improve survival of the class A MN population. The robust, beneficial effect of TF addback is consistent with reports that TFs can salvage unhealthy neurons in animal models of ALS and have a neuroprotective effect on axotomized MNs (Gimenez y Ribotta et al., 1997; Sendtner et al., 1997).

Changes that occur early in neurodegenerative processes might provide key, clinically relevant insights into how to identify cells that possess the capacity for full functional restoration. In this study, we showed that MNs exposed to stress-inducing conditions rapidly exhibited changes in their neurite length and number of nodes. Changes in these parameters appeared to occur early in the death process and constituted sensitive indices of neuronal health. Neurite retraction and reduced soma size are known to accompany neurodegeneration, even in patients (Kiernan and Hudson, 1993; Anglade et al., 1997; Takeda et al., 2014). We therefore divided MNs into classes based on their number of nodes and used single-cell tracking to examine changes in node number and correlate them with survival outcomes. We observed that neurons with more nodes had a higher survival rate. We further found that cell body size is another strong predictor of survival outcome. However, of these two early morphologic events, the number of nodes is the more feasible to track temporally in single cells. Our findings illustrate the utility of novel analytical approaches that can be applied to live-cell imaging data sets, especially when compared to more conventional types of survival assays.

Our live-cell analysis platform possesses flexibility and analytical power, making it broadly applicable to a wealth of cell biology inquiries. Live-cell analysis of individual cells is also strongly complemented by the current growing trend in single-cell RNA sequencing (Chen and Arlotta, 2016; Shekhar et al., 2016; Zheng et al., 2017). These technologies can be used to study cellular processes that occur during disease progression and determine how treatments affect these processes or others that underlie disease states and precede cell death. These studies may also help elucidate ideal time points for optimal therapeutic intervention. Hopefully, observations like these will lead to the development of a new generation of therapeutics capable of truly reversing degenerative phenotypes.

## Synthesis

Reviewing Editor: Maiken Nedergaard, University of Rochester Medical Center Decisions are customarily a result of the Reviewing Editor and the peer reviewers coming together and discussing their recommendations until a consensus is reached. When revisions are invited, a fact-based synthesis statement explaining their decision and outlining what is needed to prepare a revision will be listed below. The following reviewer(s) agreed to reveal their identity: kristen brennand, Lori Buhlman.

The reviewers and i are in agreement that is an important and clearly presented study. Minor revision is recommended. The reviewers commest are:

Reviewer 1:

This is an excellent and straightforward report that presents a novel methodology that will be helpful to hiPSC-based studies of ALS and an approach that will be widely applicable to hiPSC-based models of many neuropsychiatric disorders.

“All experiments were confirmed to have similar efficiencies of MN differentiation, even distribution of Islet-postive cells and high imaging quality. A two tailed t-test was used to determine the statistical significance between experimental conditions.”

How was the distribution of Islet-positive cells assessed?

The authors have prepared a clear and concise manuscript entitled “Using automated live cell imaging to reveal early changes during human motor neuron degeneration.” They differentiated a HUES8-Islet1::GFP cell line to motor neurons in 2D and 3D cultures, across a range of conditions, in order to optimize live cell imaging of single motor neurons across time and during neurodegeneration induced by trophic factor withdrawal (and subsequent restoration). With minor revisions, this manuscript will be suitable for publication in eNeuro.

Minor revisions

1. Methodology - Can the authors clarify which methodology (2D vs 3D differentiation, starting cell densities, etc where used in each of the five independent batches of MNs described in the methods section ‘Motor neuron production’ (seemingly all results were from 3D differentiations at 35K seeding density, but it might be helpful to present the methodological validation done as part of assay development)? Can they clarify whether the results presented in Figures 1-5 aggregate results across all five experiments? Could inter-experiment findings be presented side-by-side in an SI figure?

2. Cell line and cell-type variability - All work seems to have been conducted in HUES8-Islet1::GFP MNs. Can the authors speculate on how sensitive, if at all, their methodology might be across ESC/IPSC lines and neuronal subtypes?

a. The impact of this report could be dramatically extended by testing the cell-type-specificity of the findings. For example, could the authors also consider Islet1-GFP negative neurons (by repeating the experiment with a constitutive LV-SYN1-mCherry reporter, for example)?

b. Another interesting experiment, although likely beyond the scope of the experiment, would be to apply a more ALS specific stress (ie. co-culture MNs with SOD1-astrocytes or SOD1-astrocyte conditioned media?

3. To verify the fidelity of the automated analysis, can the authors test any part of the quantification in Figure 1 head-to-head with manual (NIH Image J?) and/or other automated analyses (Metamorph)?

4. Can the authors please note how the image analysis software will be made publically available?

5. “All experiments were confirmed to have similar efficiencies of MN differentiation, even distribution of Islet-postive cells and high imaging quality. A two tailed t-test was used to determine the statistical significance between experimental conditions.” How was the distribution of Islet-positive cells assessed?

Reviewer #2

The authors have developed a method for time-lapsed imaging and analyses of individual differentiated human motor neurons. This work is significant, since most studies to date utilize fixed-time point studies of individual differentiated cells. This approach facilitates observation and monitoring of degeneration and recovery. It also allows the comparison of the effects of interventions at various time points. The authors described various morphological features of degenerating and recovering motor neurons that could be tracked over time, and were able to use these features to predict whether intervention with trophic factors or kenpaullone could maintain or restore morphological integrity. These methods and results are not only useful for studying pathophysiology and testing ALS therapeutics, they could be employed in observing other neuron subtypes such as dopaminergic and glutamatergic in the study of Parkinson's or Alzheimer's diseases, respectively.

Thus, this work is highly impactful, and its application could be useful in many other realms. The data appear to be sound, but there are serious discrepancies between what is depicted in some graphs and what is written in the results section. The manuscript requires revisions that are minor but critical to its acceptance for publication.

Do dotted black vertical lines in figures 1B and 1D indicate statistical significance? If so, describe how analyses were done.

It is not clear how statistical analyses were done for data in figure 2D, E, H, I. If differences are statistically significant, indicate on graph and in methods section. If not, revise claims in corresponding results section.

This work is highly impactful, and its application could be useful in many other realms. The data appear to be sound, but there are serious discrepancies between what is depicted in some graphs and what is written in the results section. The manuscript requires revisions that are minor but critical to its acceptance for publication. Here are my comments:

Critical points:

• Methods section:

o Authors should describe use of kenpaullone and indicate when TF was removed and reintroduced for each aim.

o Do dotted black vertical lines in figures 1B and 1D indicate statistical significance? If so, describe how analyses were done.

• Results section:

o Indicate time point at which data for Figure 1F were taken.

o If p = 0.0231 for neurite length (Figure 1G), authors should use the standard indication for degree of significance for when p < 0.05, ‘*.’ Data for number of nodes has a similar discrepancy between Figure 1G and corresponding results section.

o How were statistical analyses done for data in figure 2D, E, H, I? If differences are statistically significant, indicate on graph and in methods section. If not, revise claims in corresponding results section.

o If p = 0.012 for the effect of kenpaullone on MN number (Figure 2G), authors should use the standard indication for degree of significance for when p < 0.05, ‘*.’

o Results section indicates that the number of MNs with more than 4 nodes increases throughout the imaging period in TF+ condition; however, Figure 3B does not depict this. Rather, the number looks to be rather stable and perhaps slightly decreased. Please address this.

o Authors claim that compared to TF+, in TF-, the population of MNs with 4 or more nodes was significantly smaller (Figure 3B,C). Please add a histogram to Figure 3 showing this comparison, including the time point at which this analysis was made. Describe statistical analysis in methods section.

o Results section indicates that TF-/Ken5uM is significantly larger than TF- (Figure 3F). Figure 3F does not depict this. Revise.

o Description of Figure 4E indicates that only TF-/Ken5uM was increased compared to TF-; however, the graph in 4E shows that TF-/Ken2.5uM also was increased compared to TF-. Clarify.

Figure 1A: Images in lower panel (TF-) should be better described. Is the first panel taken at the same time point as its counterpart in the upper panel? If so, indicate, ‘Day1,’ or the correct reference to the time point depicted in the top left image. Revision should include some indication of time point depicted in lower middle and right panels.

Figure 2A: For the second ‘Day 0,’ indicate as ‘Day 1,’ as done in 1F. I suggest removing analysis window indicators; graphs indicate this information. The AWs add a bit of confusion here.

Figure 2D,E,H,I: Add indications of significance if relevant.

Figure 3D: Indicate how soon after Day 0 analysis began, and remove indication for ‘Analysis window.’

Figure 3E: Results section indicates that only 6dTF+ increase node number compared to TF-; however, the figure indicates that two addback conditions increased node number. Clarify. Further, it is not clear to which histogram bars statistical line indicators are referring.

Figure Legend 3D: Indicate when analysis window began. Was it consistent between experiments?

Figure Legend 4 A,B: These graphs do not show results of statistical test; therefore, descriptions these graphs should be qualitative only.

Figure Legend 4A,B: Clarify whether TF was withdrawn at day 1, as indicated previously, or at day 6 as indicated in the legend.

Figure Legend 4 C and D needs to be rewritten to better describe graphs.

Minor points:

Are the automated live time-lapse imaging instrument (ALCI), and imaging software publicly available?

Visual abstract: Define ‘TF’ (Trophic Factor).

Introduction

• ‘in vitro’ should be italicized throughout.

• Authors should mention the rationale for use of kenpaullone.

• Authors should describe, ‘node,’ and the significance of node number to neuronal health.

Methods section

• Time-lapse live imaging:

o missing a closing parenthesis at the bottom of page 6.

o ‘Morphological filters’ needs to be better described. How were these filters generated? What are their upper and lower thresholds for each measurement?

• Cell Tracking:

o Figures 4,5 do not depict tracking analysis method as suggested here. Revise.

o Indicate how many cells were tracked.

o Last sentence of the first paragraph should read ‘objects,’ rather than ‘objected.’

• Statistical analysis:

o Here it is indicated that there were 5 biological replicates. In ‘Time-lapse live imaging section’ of Methods, it is stated that at least 3 biological replicates were used. Please clarify.

Results:

• Revise ‘because since,’ in last sentence of first paragraph.

• For description of data in Movie 3, indicate panel number with condition.

• Be consistent when referring to media data (i.e., indicate, ‘Movie,’ or ‘Video.’).

• Figure 4E comparison description in results section is missing a p value.

• For description of data in Figure 5, both p values do not reflect what is shown in 5C,D. Update so that p values in results section reflect significance star indicators in 5C,D.

Figure 2B: Indicate ‘Day 6’ for TF Addback images in the middle row.

Figure 2F: remove AW1 information.

Figure 3E: There is no indication to what ‘AW2’ box is referring; please remove it.

Figure 3F: Remove ‘AW1’ box.

Figure 4: Remove ‘AW’ from boxes. ‘Day6 to day14,’ and ‘day0 to day14,’ are sufficient.

Figure 5: Add ‘Day7, Day8’ to ‘Common features...’ box.

Figure 5B: The ‘2’ in ‘mm2’ should be superscripted.

Table 2 Title repeats idea: ‘tracked,’ and ‘tracking.’

Figure Legend 2: Some comparisons reached a significance of p < 0.001 (***). Add this information to the bottom of the legend.

Figure Legend 3 E and F: Remove references to AW1,2.

Figure Legend 4: Indicate what ‘***’ represent.

Multimedia/Extended Data - Movie 3: Describe conditions in more detail. For example, for conditions B and C, indicate whether TF was removed.
